# Radiation exposure of Staff handling ^18^Fluorine-Fluorodeoxyglucose in a new positron emission tomography/computed tomography centre

**DOI:** 10.4102/hsag.v28i0.2211

**Published:** 2023-07-20

**Authors:** Lerato Mosima, Nathaniel Muzamhindo, Maryke Lundie, Beverley Summers

**Affiliations:** 1 Department of Pharmaceutical Sciences, School of Pharmacy, Sefako Makgatho Health Sciences University, Pretoria, South Africa

**Keywords:** occupational radiation exposure, ring dosimeter, thermoluminescent dosimeter, polimaster pocket dosimeter, PET/CT facility, ^18^Fluorine-Fluorodeoxyglucose, radiopharmacists, radiographers

## Abstract

**Background:**

Positron emission tomography/computed tomography (PET/CT) is an imaging modality that combines images from high-energy gamma rays emitted by a positron emitting radiopharmaceutical and those from the CT component. The images are then used in the diagnosis of severe diseases. Procedures with PET radiopharmaceuticals introduce a risk of high occupational radiation exposure to staff handling them. ^18^Fluorine-Fluorodeoxyglucose ([^18^F]FDG) is the most commonly used PET radiopharmaceutical.

**Aim:**

To determine the radiation exposure of staff working at the PET/CT facility.

**Setting:**

Academic hospital in Gauteng.

**Methods:**

The study was quantitative and descriptive. The radiation exposure data of participants were collected using Polimaster^®^electronic pocket dosimeters, ring dosimeters and thermoluminescent dosimeters. The participants’ workflow was tracked and the tasks that led to the highest radiation exposure were identified.

**Results:**

Radiopharmacists had 129 dispensing days with the resultant daily radiation exposure ranging between 0.01 µSv and 0.32 µSv. The radiographers’ daily radiation exposure ranged between 7.08 µSv and 19.14 µSv. Radiographers received the highest radiation dose during radiopharmaceutical injection (average = 1.86 µSv).

**Conclusion:**

The study found that staff working at a new PET/CT facility in Gauteng were not at risk of radiation exposure above the accepted annual limits, which are 20 mSv per annum, averaged over 5 years, and with no more than 50 mSv in 1 year.

**Contribution:**

The findings revealed the need for continuous training in radiation protection measures for all staff working in the PET/CT facility.

## Background

Nuclear medicine departments employ advanced imaging technologies such as a hybrid of positron emission tomography (PET) and computed tomography (CT) for diagnosis and therapy of many diseases (Ramamoorthy 2018). Positron emission tomography and computed tomography is now the most increasingly desired imaging modality for a number of oncological diseases because it offers both functional and morphological data of patients’ conditions (Al-Aamria, Al-Balushia & Bailey 2019; Dalianis et al. 2014). Positron emission tomography and computed tomography radionuclides emit high-energy ionising radiation. This type of radiation can ionise matter due to its energy being greater than the ionising potential of matter (Bailey et al. 2014).

^18^Fluorine-Fluorodeoxyglucose ([^18^F]FDG) is the most commonly used radiopharmaceutical in PET/CT facilities. Radiopharmaceuticals are medicinal products that comprise of a radionuclide and a biological component that carries the radionuclide to the target organ or cells; they are prepared in a radiopharmacy unit attached to a nuclear medicine department (Ramamoorthy 2018). During decay of PET radiopharmaceuticals, a positron is emitted from the nucleus of the radionuclide and interacts with an electron in the human tissue, causing an annihilation reaction, which results in two gamma photons travelling in opposite directions at an angle of 180°, each with an energy of 511 keV (Ziegler 2005). The specific gamma ray constant of fluorine-18 is almost six times more than that of technetium-99m, hence stringent radiation protection measures need to be in place (Al-Aamria et al. 2019). Radiation workers handling radiopharmaceuticals or patients injected with radiopharmaceuticals are at risk of occupational radiation exposure and developing adverse biological effects, such as cancer, due to changes in cell DNA caused by this type of radiation (Bailey et al. 2014). Non-cancer health effects of ionising radiation include skin burns, cataracts and infertility, each of which differs greatly depending on the radiation dose and response of tissue to radiation, that is, tissue threshold (Hamada & Fujimichi 2014).

To ensure effective radiation protection of radiation workers in South Africa, the South African Health Product Regulatory Authority (SAHPRA) enforces the annual limits on radiation exposure as given by the International Atomic Energy Agency (IAEA) and the International Commission on Radiological Protection (ICRP) (SAHPRA 2022; IAEA 2018) which are as follows:

- 20 mSv dose per year averaged over 5 years (100 mSv in 5 years) and 50 mSv in any single year.
- 20 mSv equivalent dose per year to the lens of the eye, averaged over 5 years (100 mSv in 5 years) and 50 mSv in any single year.
- 500 mSv equivalent dose per year to the extremities (hands and feet) or to the skin.

Personnel working in the PET/CT facility should wear radiation monitoring devices at all times to ensure that the radiation dose received falls within the set annual limits (Bailey et al. 2014). Individual radiation monitoring dosimeters measure the effective body radiation dose using either a chest thermoluminescent dosimeter (TLD) that gives the cumulative radiation exposure dose or an active personal dosimeter that gives real-time radiation measurements. The radiation exposure to the extremities is measured using a finger/ring dosimeter (Taha, Shahein & Hassan 2008). Minimising occupational exposure to radiation requires ensuring that the ALARA (As Low As Reasonably Achievable) principles are adhered to and includes the following precautions:

- Spending as little time as possible with the radioactive source (the [^18^F]FDG dose/vial or the patient injected with the radiopharmaceutical), that is, planning tasks beforehand and practising before executing the task as to minimise mistakes.
- Increasing the distance between the personnel and the radioactive source.
- Shielding, for example, includes the use of lead shielding and lead glass during radiopharmaceutical dose drawing and dispensing, use of lead syringe shields during radiopharmaceutical injection and use of lead-shielded carriers when transporting radioactive sources (Donmoon, Chamroonrat & Tuntawiroon 2016).

The PET/CT facility where the current study was conducted opened in June 2017, with a dedicated PET radiopharmacy as part of the installation. The facility had a full staff complement of nuclear medicine consultants, radiographers, nurses, medical physicists and radiopharmacists. Radiographers and radiopharmacists in this new PET/CT facility were the only two groups of professionals who handled [^18^F]FDG or patients injected with it. However, most of these radiographers and radiopharmacists had limited experience in working with high energy PET radiopharmaceuticals. A need was therefore identified to measure the occupational radiation exposure of radiographers and radiopharmacists, by observing the handling process of [^18^F]FDG during their workflow as well as determining the work processes that lead to high radiation exposure to this group. The aim and objectives of this study were to determine the radiation exposure of radiographers and radiopharmacists working at the PET/CT facility in a hospital in Gauteng (with the use of TLDs, ring dosimeters and electronic pocket dosimeters) and to identify tasks and work practices which led to the highest radiation exposure to this group.

## Methodology

### Study design

The study was quantitative and descriptive in nature. Radiation exposure data of participants were collected from the inception of the PET/CT facility in June 2017 to September 2018.

### Setting

The study was conducted at the PET/CT facility of an academic hospital in Gauteng, South Africa. ^18^Fluorine-Fluorodeoxyglucose was the only PET radiopharmaceutical used at the time of the study. The [^18^F]FDG was ordered a day in advance, based on the number of patients, the patients’ weights and scheduled time of injection for each patient. The doses were either delivered as single or bulk doses.

### Study population

The study population included all the staff members working in the PET/CT facility who handled the radiopharmaceutical ([^18^F]FDG) or who dealt with patients injected with the radiopharmaceutical, that is, five radiographers and eight radiopharmacists who were allocated to work in the PET/CT facility at the time of the study. The gender distribution of participants was as follows: two male radiographers, three female radiographers, two male radiopharmacists and six female radiopharmacists. It was noted that 78.78% of the female participants were of child bearing age. Radiation exposure data for all radiographers and radiopharmacists who worked in the PET/CT facility during the study period was collected. Other staff who worked in the PET/CT facility during the study period (e.g. nurses, nuclear medicine physicians, medical physicists and cleaners) did not handle the radiopharmaceutical or the patients injected with the radiopharmaceutical and were therefore excluded from the study.

### Data collection

**FIGURE 1:**
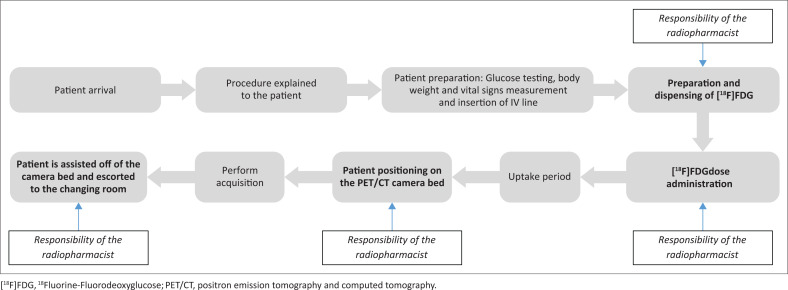
Workflow in the positron emission tomography and computed tomography facility of a hospital in Gauteng.

The workflow of radiographers and radiopharmacists in the PET/CT facility and their involvement in the different steps of the patient handling process for [^18^F]FDG are depicted by Figure 1. The events shown in boldface letters carry the highest possible radiation exposure due to longer interaction with patients injected with [^18^F]FDG or direct interaction with the radiopharmaceutical itself. The staff members responsible for each of these events are indicated in italics.

Participants were given three radiation exposure monitoring devices to wear exclusively in the PET/CT facility during the study with the exception of radiographers, who only wore two radiation exposure monitoring devices exclusively in the PET/CT facility. Radiographers rotated between the PET/CT facility and the single photon emission computed tomography (SPECT) facility and wore the same TLDs in both the SPECT and PET/CT facilities. Therefore, the radiation exposure received from their work in the PET/CT facility could not be differentiated from their radiation exposure in the SPECT facility. The radiographers’ radiation exposure data from TLDs was therefore not included in the study. Radiographers were scheduled to the PET/CT facility in pairs on a weekly rotational basis while radiopharmacists were allocated once a week.

The radiation measuring devices were:

- Ring dosimeters (issued by the South African Bureau of Standards [SABS]) that measured the total radiation dose to hands over a predetermined period of time. These were marked according to each participant’s number and issued to them on a daily basis for the entire duration of the study. At the end of each shift, the participants returned the ring dosimeters to the researchers for safe keeping. The participants wore the ring dosimeter on the preferred finger of their dominant hand throughout the study.
- Thermoluminescence dosimeters (issued by the SABS) that measure the total whole-body radiation dose received over a predetermined time were issued to participants on a monthly basis and they wore their TLDs on their upper chest outside their laboratory coats.
- Polimaster^®^ PM1610 electronic pocket dosimeters that measured the whole-body radiation dose received per minute per task were issued to participants on a daily basis and the device number documented. The Polimaster^®^ PM1610 electronic pocket dosimeters were never reset at the end of the day; however, the dose in the morning and at the end of the day were recorded. Therefore, the acquired dose would be the difference between the dose in the morning and the dose at the end of the day. All participants wore laboratory coats with a pocket on the upper right chest, and this is where they clipped their Polimaster^®^ PM1610 electronic pocket dosimeters.

The researchers adapted four data collection tools from the ones used by Lundie, Summers and Kemp (2017) in a similar study conducted at a private hospital in Gauteng:

- Patient cross-reference list: It recorded the patient number and the dosage (activity) the patient received.
- Work flow tracking form: It recorded the tasks performed by participants and the time it took to complete the tasks. The researcher observed all tasks performed by participants and would record the time a participant begins a task and the time the participant finishes the task. The time it took to complete a single task would then be linked to the radiation exposure retrieved from the Polimaster^®^PM1610 electronic pocket dosimeters.
- Personal radiation dose measurement form: It recorded the radiation exposure received by each participant during the study as recorded by the different dosimeters used.
- Polimaster^®^ PM1610 electronic pocket dosimeter tracking form: It recorded the Polimaster^®^ electronic pocket dosimeter serial number and the participant to whom the Polimaster^®^ pocket electronic dosimeter was issued for each day.

The researchers used the above tools and radiation exposure measurements as recorded by the different dosimeters to collect radiation exposure data for all radiographers and radiopharmacists. The researchers observed the workflow of participants and the time it took for each participant to complete a task was recorded.

### Data analysis

Data were extracted from a hard copy file kept in the PET radiopharmacy with the daily radiation doses received by each staff member measured by the Polimaster^®^ PM1610 electronic pocket dosimeters. The data was entered onto Microsoft Excel spreadsheet. The participants’ cumulative radiation exposure for the study period, as measured by their ring dosimeters, was obtained from the Radiation Protection Service of the SABS and was transferred to Microsoft Excel spreadsheets for assessment of total radiation exposure of each participant for the period of the study. The daily log of patients seen and the activity (dose) administered to each patient was also recorded on the spreadsheet.

As the radiographers working in the PET/CT facility are involved in various steps/tasks in the patient handling process (see Figure 1), a workflow tracking data collecting form was used to record the time it took them to complete every task in the patient handling process. The data from this form was compared with the data from the Polimaster^®^ PM1610 electronic pocket dosimeters on a Microsoft Excel spreadsheet to identify participants who received the highest radiation exposure, as well as the task(s) that led to higher radiation exposures. The data from the Polimaster^®^ PM1610 electronic pocket dosimeters worn by radiopharmacists were entered onto an Excel spreadsheet and compared to the total daily activity dispensed (radiation dose to be injected to patients).

### Reliability and validity

The data collection process was reliable as the systems that were employed to collect data have been used before by Lundie et al. (2017) in a similar study at a private hospital in South Africa. The data collected with the ring dosimeters was valid as the ring dosimeters were calibrated by the SABS. The Polimaster^®^ PM1610 electronic pocket dosimeters were newly acquired and calibrated by the manufacturer before their start of use in June 2017.

### Ethical considerations

This study was approved by the Research Ethics Committee of a university in Gauteng (Research number/P/267/2017:PG and Research number/P/236/2018:PG). Permission to conduct the study at the PET/CT facility of the academic hospital in Gauteng, South Africa, was obtained from the clinical manager of the hospital and the Nuclear Medicine Head of Department. The participants gave written, informed consent to have the researcher observe the patient handling process carried out by radiographers and the ^18^F-FDG handling by radiopharmacists. Patients’ privacy was ensured throughout the study as their personal details were not recorded on any of the data collecting tools used by the researcher.

## Results

The results of the radiation exposure of radiographers and radiopharmacists will be presented under the following titles: ‘Radiographers’ occupational radiation exposure in the PET/CT facility’ and ‘Radiopharmacists’ occupational radiation exposure in the PET/CT facility’. Under these headings, the mean radiation exposure, radiation exposure related to the radiopharmaceutical dose handled, the types of radiopharmaceutical packages, radiation exposure per task and the radiation exposure to hands, will be described. A total of 134 dispensed [^18^F]FDG doses were recorded successfully during the study. ^18^Fluorine-Fluorodeoxyglucose was delivered daily as either bulk vials (eight doses), individual syringes for each patient scheduled for the day (73 doses), or syringes containing bulk doses that had to be manipulated to dispense the required dose for each patient for the day (53 doses).

### Radiographers’ occupational radiation exposure in the positron emission tomography and computed tomography facility

#### Mean radiation exposure of radiographers

Table 1 shows the relative radiation dose received by each radiographer, as measured by their Polimaster^®^ PM1610 electronic pocket dosimeters.

**TABLE 1:** Mean radiation exposure received by each radiographer in the positron emission tomography and computed tomography facility measured by the Polimaster^®^ PM1610 electronic pocket dosimeters.

Radiographer number	Wearing period in days	Mean radiation exposure (µSv)
1	21	7.74
2	21	7.07
3	38	14.04
4	20	7.49
5	20	19.14

The mean radiation exposure of radiographers as measured by the Polimaster^®^ PM1610 electronic pocket dosimeters ranged between 7.07 µSv and 19.14 µSv, which is below 1 mSv (1000 µSv). A daily dose of 1 mSv is considered the minimum dose at which stochastic effects may occur. Stochastic effects are defined as the random or probabilistic genetic changes or carcinogenesis that may occur due to radiation exposure (Peck & Samei 2017).

Radiographer 5 received the highest average radiation exposure per day (19.14 µSv), while the lowest dose was received by radiographer 2, who only received a mean radiation exposure of 7.07 µSv. However, even if radiographer 5 who received the highest radiation dose, received the same dose every day, the radiographer’s combined annual dose would still be below the prescribed annual dose limits.

The annual radiation dose of radiographer 5 was extrapolated to demonstrate the worst-case scenario, that is, highest occupational radiation exposure possible for this radiographer. The calculation below was based on the fact that the PET/CT facility operated from Monday to Thursday every week during the study period, which resulted in a 4-day working week.

19.14 µSv × 4 working days per week × 2 weeks per month = 153.12 µSv per month

153.12 µSv per month × 12 months in a year = 1837.44 µSv ÷ 1000 = 1.837 mSv per year

The extrapolated radiation exposure dose of 1.8 mSv is below the annual dose limits stipulated by the ICRP which is 20 mSv per year averaged over 5 years (100 mSv in 5 years) and 50 mSv in any single year (Bailey et al. 2014).

#### Radiographer radiation exposure per task in the positron emission tomography and computed tomography facility workflow

**FIGURE 2:**
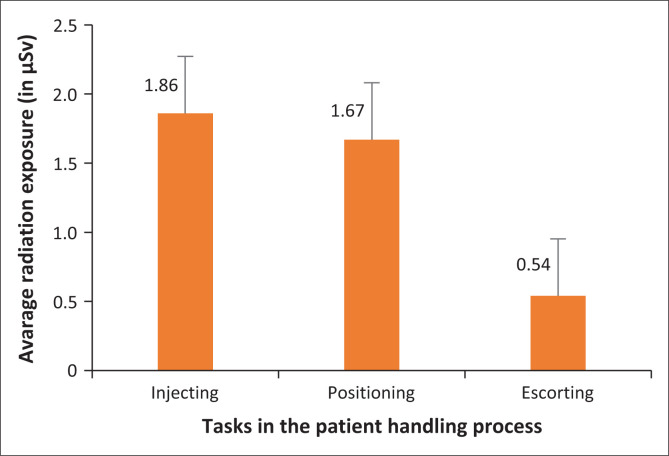
Average radiation dose (in µSv) per task in their workflow as measured by the Polimaster^®^ PM1610 electronic pocket dosimeters.

The patient handling process at the PET/CT facility is divided into three tasks in which the radiographers are involved and which have the potential of occupational radiation exposure: radiopharmaceutical injection, patient positioning on the PET/CT camera bed and patient escorting from the camera bed to the change room. Figure 2 illustrates the average radiation exposure of each radiographer during the execution of each task in the patient handling process.

The task in the patient handling process that resulted in the highest radiation exposure for radiographers was radiopharmaceutical injection, with an average of 1.86 µSv ± 1.87 µSv for all the radiographers. This could be due to the fact that when radiographers inject the patient they get exposed to high activity as opposed to the other tasks that allow for decay of the ^18^F-FDG over time, resulting in low radiation exposure. The task that led to the second highest occupational radiation exposure overall is positioning of the patient on the PET/CT camera bed (1.67 µSv ± 1.05 µSv), due to prolonged time spent with the injected patient, especially when the patient is not mobile and requires direct assistance (Donmoon et al. 2016). The last task in the patient handling process, which is escorting the patient off the camera bed to the change room, led to the least radiation exposure (0.54 µSv ± 0.73 µSv) as very little time is spent with the patient and one half-life of the radiopharmaceutical has decayed to half of the injected dose (Al-Aamria et al. 2019).

#### Radiation exposure of radiographers related to the injected activity of ^18^Fluorine-Fluorodeoxyglucose

**FIGURE 3:**
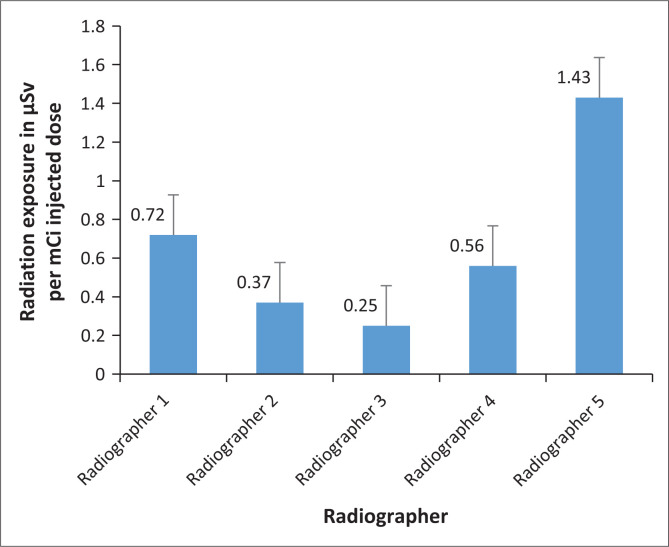
Average radiation exposure (in µSv) of radiographers, per mCi injected dose of ^18^Fluorine-Fluorodeoxyglucose as measured by the Polimaster^®^ PM1610 electronic pocket dosimeters.

Figure 3 presents the radiation exposure of radiographers per mCi injected dose of ^18^F-FDG as measured by the Polimaster^®^ PM1610 electronic pocket dosimeters.

Radiographer 5 received the highest exposure per injected activity (1.43 µSv/mCi) as compared to other radiographers who received a mean exposure/activity below 1 µSv/mCi. From observation, radiographer 5 had to assist other radiographers with their tasks; this may explain the high radiation exposure the radiographer received. Radiographer 5 was one of the two radiographers who had previously worked in a PET/CT facility and therefore had more experience.

The average radiation exposure doses received by radiographers per injected activity of [^18^F]FDG were lower than the prescribed annual limits. This may be due to the routine use of syringe shields during radiopharmaceutical administration, separate injection rooms for patients in the PET/CT facility, and the use of Polimaster^®^ PM1610 electronic pocket dosimeters with alert tones that increase the awareness of radiographers when they are exposed to higher levels of radiation that exceed the limit set on the device.

#### Radiographer radiation exposure to the hands

**FIGURE 4:**
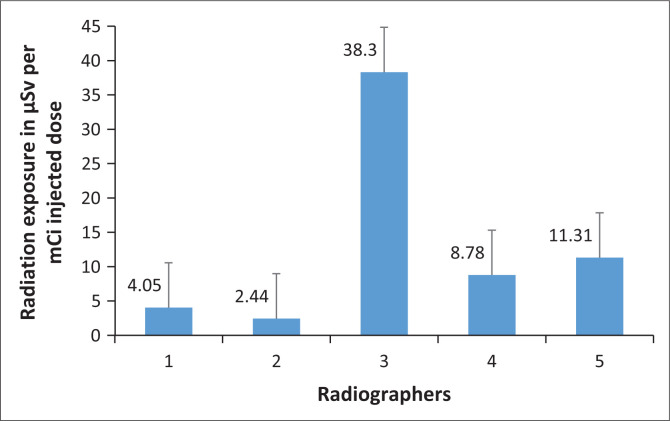
Injected activity-related radiation exposure to the hands of radiographers as measured by ring dosimeters (in µSv per mCi activity injected).

Figure 4 presents the radiation dose to the hands received by each radiographer, as measured by the ring dosimeters.

The radiation dose per activity injected to the hands of radiographers ranged between 2.44 µSv and 38.30 µSv. Radiographers 1 and 2 had the lowest radiation exposure to their hands, while radiographer 3 received the highest radiation exposure. From observation, radiographer 3 received higher radiation exposures because this radiographer had to assist other radiographers with the task of radiopharmaceutical injection.

The maximum radiation exposure doses to the hands of each radiographer as measured by the ring dosimeter were 140 µSv for both radiographers 1 and 2, 1390 µSv for radiographer 3, 540 µSv for radiographer 4 and 340 µSv for radiographer 5.

### Radiopharmacists’ occupational radiation exposure in the positron emission tomography and computed tomography facility

#### Radiopharmacists’ average whole body radiation exposure

The radiation dose for all radiopharmacists as measured by their TLDs over the entire study period was received from SABS and reported as zero radiation dose, with the exception of June 2017, August 2017 and January 2018, where TLD radiation exposure results could not be found. However, it is important to note that the zero report is not a true zero, but a representation of radiation exposure doses below 0.15 mSv. All values that are below the threshold of 0.15 mSv (150 µSv) are reported as zero by SABS, as these doses are comparable to background radiation.

**FIGURE 5:**
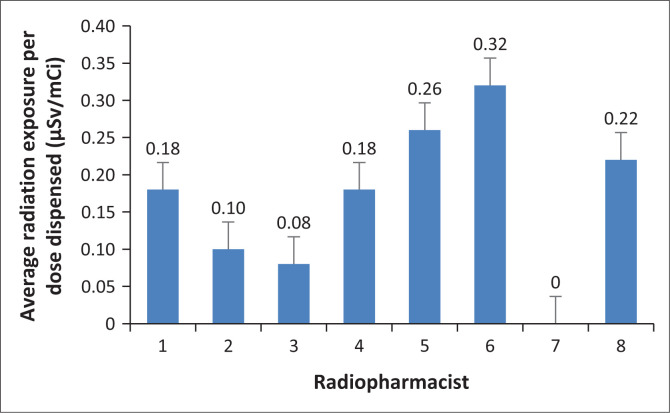
Average daily radiation exposure of radiopharmacists per mCi dose dispensed (μSv/mCi).

The average whole-body radiation exposure per mCi [^18^F]FDG activity dispensed is shown in Figure 5.

**FIGURE 6:**
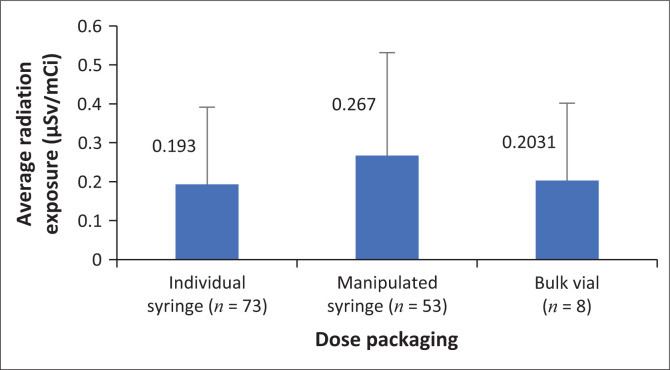
Average daily radiation exposure per mCi ^18^Fluorine-Fluorodeoxyglucose ([^18^F]FDG) dose dispensed (μSv/mCi) for the three types of [^18^F]FDG packaging.

The majority of radiopharmacists had relatively low radiation exposure. From observation, the higher radiation exposure received by radiopharmacists 5 (0.26 µSv/mCi), 6 (0.32 µSv/mCi) and 8 (0.22 µSv/mCi) could be ascribed to their limited experience in dispensing radiopharmaceuticals, as these three radiopharmacists were newly employed. Al-Amaria et al. (2019) stated that the occupational radiation exposure received by personnel differs based on the skill of the personnel. When data from all the radiopharmacists were combined, the average annual Polimaster^®^ PM1610 electronic pocket dosimeter reading per mCi dispensed over the duration of the study was 0.19 µSv/mCi for dispensing from individual syringes compared to 0.27 µSv/mCi for manipulated bulk syringes. The average radiation exposure for radiopharmacists per mCi [^18^F]FDG activity dispensed from the three types of packaging that the radiopharmaceutical is supplied in is illustrated in Figure 6.

The radiation exposure was lowest at 0.19 µSv/mCi when individual dose packaging was used. This suggests that the use of individual dose packaging is beneficial in facilitating low radiation exposure per mCi dose dispensed. Dispensing of [^18^F]FDG from bulk vials also led to relatively low radiation exposure (0.20 µSv/mCi) to radiopharmacists. From observation, it was ascertained that the PET/CT radiopharmacy had a lead-shielded dose drawing system designed for dispensing from a bulk vial. The highest radiation exposure (0.26 µSv/mCi) was received from manipulating bulk syringes to obtain doses for specific patients.

## Discussion

Table 1 presents the mean radiation exposure received by radiographers in the PET/CT facility. The highest radiation exposure was 19.14 µSv for radiographer 5. Although this was the highest radiation exposure received, when extrapolated to demonstrate the worst-case scenario of radiation exposure, the resultant radiation exposure is still below the annual dose limits described by SAHPRA. The extrapolated dose is 1837.44 µSv or 1.84 mSv per annum which is approximately 11 times lower than the average annual dose limit of 20 mSv per annum. Therefore, there is very little risk that this radiographer would receive occupational radiation exposure above the set limits.

Figure 2 presents the tasks in the patient handling procedure that are performed by radiographers. The tasks leading to the highest radiation exposure for radiographers were identified as radiopharmaceutical injection, which had an overall mean of 1.86 µSv, and patient positioning with an overall mean of 1.67 µSv. Figure 4 revealed radiographer 3 as the radiographer with the highest radiation exposure to hands (38.30 µSv). This radiographer was observed assisting other radiographers with [^18^F]FDG injection to patient; this further supports the finding in Figure 2, that radiopharmaceutical injection carries the highest risk of occupational exposure. Another study conducted locally by Lundie et al. (2017) to measure the radiation exposure of radiographers who handled [^18^F]FDG in a PET/CT facility at a private hospital in South Africa discovered that radiographers received the highest radiation doses during patient injection with [^18^F]FDG, weighing the patient and measuring glucose levels in the presence of radioactive patients and with prolonged time spent with injected patients during patient positioning and scanning. In this study, escorting patient to the change room carried the lowest radiation exposure risk with radiation exposure of 0.54 µSv. Escorting patients to change rooms is one task that can be carried out safely by a pregnant radiographer with foetal radiation exposure kept at its minimal. The ICRP has set the radiation exposure of pregnant radiation workers at 1 mSv over the entire term of pregnancy (Al-Aamria et al. 2019).

Figure 3 represents the average radiation exposure of radiographers per mCi dose of [^18^F]FDG. These data simply confirm the data in Table 1 with radiographer 5 having received the highest radiation exposure. In Figure 3, the highest radiation exposure is 1.43 µSv, received by radiographer 5. This solidifies the fact that more time spent in the presence of or handling patients injected with [^18^F]FDG increases the risk of occupational exposure (Donmoon et al. 2016).

The only task in the patient and radiopharmaceutical handling process where radiopharmacists are at risk of radiation exposure is the dispensing of [^18^F]FDG. Figure 5 demonstrates that the highest average daily radiation exposure during dispensing of [^18^F]FDG was 0.32 µSv/mCi. Manipulation of bulk syringes to obtain individual patient doses results in the highest radiation exposure to radiopharmacists as revealed by Figure 6. This task results in an overall daily mean radiation exposure of 0.267 µSv/mCi dose dispensed. These results are similar to the findings of an international study conducted by Donmoon et al. (2016) to measure the radiation exposure of Nuclear Medicine staff during [^18^F]FDG procedures at Ramathibodi Hospital, Bangkok, which concluded that radiopharmacists received higher radiation exposure doses when drawing a dose from a bulk vial compared to the receipt of individual patient doses. All the findings of this study revealed that the radiation exposure received by staff at this facility does not exceed the radiation exposure limits set for radiation workers.

Because there is no threshold below which no biological effects occur, any radiation dose received has the potential to cause harm (Lundie et al. 2017). Staff exposed to radiation at this facility can therefore still employ more safety measures to ensure further reduction in radiation exposure. Some of these measures are listed below:

- Habitual use of lead shielding apparatus such as lead syringe shield for radiopharmaceutical dose drawing and injection, and the use of lead tongs when transferring the dose from the lead pot to the dose calibrator.
- Ordering of single doses as this carries the lowest radiation exposure risk (Figure 6), because radiopharmaceutical dose drawing and manipulation are eliminated.
- Radiographers rotating radiopharmaceutical injection task between patients. The researchers observed cases where one radiographer injected all the patients, positioned them on the PET/CT camera bed and escorted them to the change room, while the other radiographer operated the PET/CT camera and was therefore not exposed to any radiation from the radiopharmaceutical itself or the radioactive patient. It is recommended that radiographers have a set Standard Operating Procedure manual that states that one radiographer handles the first patient and the other operates the PET/CT camera, after which the radiographers swap roles for the next patient to minimise the radiation exposure that would be received by one radiographer if he/she were to handle all the patients scheduled for a specific day.
- Initial and continuous internal refresher training on radiation protection measures such as application of the ALARA principle, proper use of lead shielding material, separation of patient injected with radiopharmaceuticals from those not injected, maintenance of safe distance from radioactive source and proper dose drawing. Because the experience of staff varies, the more experienced staff can offer routine training on radiation safety measures and the ALARA principles to less experienced staff.

The limitations encountered during the conduct of the study were:

- Budget constraints: It was not possible to procure blank TLDs for radiographers to use exclusively in PET/CT. As a result, data from TLDs worn by radiographers were excluded from the study as radiographers wore the same TLD in SPECT as well.
- [^18^F]FDG production failure which led to the suppliers not delivering the expected dose and thereby cancellation of all booked cases for the day and no data collection. ^18^Fluorine-Fluorodeoxyglucose production failure happened four times during data collection, which resulted in 4 days of no data collection.
- PET/CT failure: There was one occasion during patient scanning, that the PET/CT malfunctioned and could not scan anymore, so the patient was rebooked. However, it took 3 days to fix the PET/CT that resulted in an additional 3 days of no data collection.

## Conclusion

Although the study identified injection of [^18^F]FDG and positioning of patients injected with [^18^F]FDG on the PET/CT camera bed as the tasks leading to the highest radiation exposure for radiographers and dose manipulation as the task leading to the highest radiation exposure for radiopharmacists, the findings also proved that with increased awareness to the dangers associated with exposure to such high energies as the one carried by [^18^F]FDG and adherence to the ALARA principles, personnel can work safely with or around these radiopharmaceuticals.

The main goal of this study was to measure the occupational radiation exposure of staff in the PET/CT facility. Based on the results of the study performed at the PET/CT facility, an assumption can be made that staff working in the facility generally have high awareness of the dangers associated with the high energy radiopharmaceuticals handled in the facility and employ safe work practices to reduce the amount of radiation they get exposed to. The availability of lead syringe shields, lead pots and lead syringe carriers in the PET/CT facility as well as separate injection rooms for patients in the PET/CT facility, also contributed to the safety of staff members working in this facility.

Both radiographers and radiopharmacists received radiation exposure doses below the limits set out by ICRP even when extrapolated to demonstrate the worst-case scenario. However, even with these demonstrated safe work practices, continuous training on appropriate radiation protection measures is highly recommended to further reduce occupational radiation exposure.
